# Traumatic Brain Injury, Boredom and Depression

**DOI:** 10.3390/bs3030434

**Published:** 2013-08-02

**Authors:** Yael Goldberg, James Danckert

**Affiliations:** Department of Psychology, University of Waterloo, 200 University Avenue West, Waterloo, Ontario N2L 3G1, Canada; E-Mail: ygoldber@uwaterloo.ca

**Keywords:** traumatic brain injury, boredom, depression

## Abstract

Traumatic brain injury (TBI) often presents with co-morbid depression and elevated levels of boredom. We explored the relationship between boredom and depression in a group of mild (*n* = 38), moderate-to-severe TBI patients (*n* = 14) and healthy controls (n = 88), who completed the Beck Depression Inventory and Boredom Proneness Scales as part of a larger study. Results showed that the relationship between boredom and depression was strongest in moderate-to-severe TBI patients. We explored two boredom proneness factors that index an individual’s need for external or internal stimulation. Results indicated that the need for external stimulation was the critical driver in the relation between boredom and depression. Once again, this relationship was strongest in the moderate-to-severe TBI group. These results suggest that one common factor underlying boredom and depression is the need for stimulation from the external environment and, presumably, a failure to satisfy that need—a disconnection felt most strongly in moderate-to-severe TBI.

## 1. Introduction

Traumatic brain injury (TBI) often leads to pervasive emotional and cognitive changes, including increases in boredom and depression, and decreases in motivation [1,2,3,4]. A great deal of research, including work from our own lab, has shown a consistent and strong relationship between the constructs of boredom and depression [5,6,7]. Importantly, we recently showed that although the two experiences are highly correlated (r = 0.72), they can be shown to be psychometrically distinct [6]. 

Work in our lab has also shown that boredom should not be considered a unitary construct [8]. The Boredom Proneness Scale (BPS) [5], most commonly used to evaluate susceptibility to boredom, has consistently been shown to have two prominent factors that account for the variance on this measure—the internal and external stimulation subscales. It should be noted that the factor structure of the BPS is far from uncontroversial—although many studies have sought to determine the factor structure of the BPS, the data has produced anywhere from two to eight factors [7]. However, the perceived need for either internal or external stimulation represents the two factors most commonly observed in all of these factor analytic studies [7,9]. This distinction harks back to an earlier one made by Greenson [10] between an agitated and an apathetic bored state. In short, the difference between these two states lies in their motivational status. The agitated boredom prone individual experiences boredom as an aggressively dissatisfying state—they are motivated to engage in something pleasurable and stimulating, but every attempt to do so fails to satisfy them. This discrepancy between the desire to be stimulated and a failure to extract that stimulation from the environment leads to a sense of agitation—hence, the so-called agitated boredom prone individual. In contrast, the apathetic boredom prone individual may perceive their environment to be dull and monotonous, but experiences no desire to alter that experience. In other words, the apathetic boredom prone individual sees things as unstimulating, but is not motivated to redress that fact. 

Recent work in our lab exploring the relationship between boredom and attention supports the distinction between the agitated and apathetic boredom prone subtypes. Others had already demonstrated a link between boredom and attention, suggesting that lapses in everyday attention (e.g., pouring orange juice on your cereal) and reduced mindfulness actually *lead* to both boredom and depression [11,12]. In this context, boredom (and potentially depression) arises as a consequence of a *disengaged mind*—an individual less capable of attending appropriately to their surroundings [12]. Our work found that it was the apathetic boredom prone individual who most prominently showed such lapses in everyday attention. In contrast, the agitated boredom prone individual was insensitive to errors of performance on a sustained attention task [8]. That is, the agitated boredom prone individual did not slow their reaction times on trials subsequent to an error, as would normally be expected [8,13]. This same kind of insensitivity to having made an error is evident in TBI patients [13]. Indeed, there is a wealth of research demonstrating poor sustained attention and concentration in patients with TBI [3,13,14,15,16]. It may be the case that the poor sustained attention evident in TBI will, in turn, lead to increased levels of boredom [11,12].

Given the distinct boredom subtypes discussed above, and the fact that both boredom and depression represent major impediments to rehabilitation [2,17], it is important to investigate whether the commonly observed relationship between the two affective constructs is driven primarily by the need for external or internal stimulation. The current study aimed to explore the relationship between boredom and depression following TBI, a population of patients who report experiencing high levels of both. *A priori*, we expected the moderate-to-severe TBI group to have the highest BPS scores, given prior research suggesting this population experiences elevated levels of boredom [4]. Furthermore, based on previous results [6], we expected that boredom and depression would be highly correlated in all groups. However, we hypothesized that this relationship would be strongest in the moderate-to-severe TBI group.

## 2. Method

### 2.1. Participants

Three groups of participants completed this study as part of a larger project exploring the cognitive and emotional correlates of boredom in TBI (Table 1). 

Participants in the control group were recruited from the undergraduate population at the University of Waterloo and participated for course credit. Participants in the mild-TBI group were also recruited from this population and had reported on a mass questionnaire administered to all Psychology students that they had previously experienced a concussion. Participants forming the moderate-to-severe TBI group were recruited from the Kitchener-Waterloo, Hamilton and Niagara communities and St. Michael’s Hospital in Toronto. Patients were tested in their homes or in the lab, where possible, and were compensated for their time at a rate of $10 per hour. Injury severity was determined using American Congress of Rehabilitation Medicine criteria, including Glasgow Coma Scale (GCS) scores and length of loss of consciousness (LoC), where available. For inclusion in the mild TBI group, a participant had to have a GCS score greater than or equal to 13 (range was 13 to 15) within at least 30 minutes of injury and an LoC of less than 30 minutes (Table 1). For inclusion into the moderate-to-severe TBI group, participants had to have a GCS scores of less than or equal to 12 and an LoC of greater than 30 minutes (Table 1). In addition, patients describing substantial post-traumatic amnesia (greater than 24 hours) were included in the moderate-to-severe TBI group. All procedures were approved by the University of Waterloo’s Office of Research Ethics, and all participants gave written consent prior to participating. 

### 2.2. Procedure

#### 2.2.1. Boredom Proneness

As part of a larger study, all participants first completed a series of questionnaires, including the Boredom Proneness Scale (BPS), which measures an individual’s susceptibility to experiencing boredom [9]. The 28-item scale includes statements, such as “I find it easy to entertain myself” rated on a 7-point Likert scale. Responses are summed for a total boredom proneness score, ranging from 28 to 196 (higher scores indicate greater boredom proneness). The BPS can be split into two subscales measuring the perceived need for either internal or external stimulation [7,8].

#### 2.2.2. Depression

Existence and severity of depression was indicated by the Beck Depression Inventory (BDI-II) [18], a widely used self-report measure of depression. The BDI-II is composed of 21 items, each consisting of four self-evaluative statements scored from 0 to 3. Responses are tallied to produce a total BDI-II score ranging from 0 to 63 (higher scores indicate greater levels of depressive symptoms). In individuals with a clinical diagnosis of depression, scores ranging from 0–13 reflect minimal levels of depression symptom severity, 14–19 reflect mild levels, 20–28 reflect moderate levels and 29–63 reflect severe levels [18].

**Table 1:** Participant Characteristics for the current study.

Group (N)	Age M (SD)	Age range	Years post injury	ACRM Criteria	BPS score M (SD)	Internal Scale M (SD)	External Scale M (SD)	BDI-II score M (SD)
Controls (88)	23 (1.4)	17–51	NA	NA	93.42 (28.31)	26.58 (15.3)	64.51 (19.68)	11.53 (11.62)
Mild TBI (38)	24 (9)	18–54		GCS 13–15 w/in 30 mins LOC < 30 mins	101.21 (23.12)	30.31 (4.14)	61.41 (19.51)	10.58 (9.87)
Mod-to-SevTBI (14)	36 (12.1)	18–56		GCS ≤ 12 LOC > 30 mins	106.21 (21.93)	34.51 (8.12)	68.43 (11.32)	13.36 (9.96)
Pt1	42		20	Coma several weeks	100	35	63	0
Pt2	36		30	Coma several months	75	28	50	4
Pt3	40		20	Coma 6 weeks	140	48	89	30
Pt4	56		30	LOC = 40 mins	100	16	11	8
Pt5	25		6	Coma 6 weeks	119	28	81	18
Pt6	34		14	Coma 6 weeks	123	39	18	16
Pt7	18		2	LOC, PTA 2 weeks	116	36	11	11
Pt8	54		10	Coma 2 weeks	80	21	52	10
Pt9	23		12	LOC 2 hours	143	35	102	32
Pt10	39		< 1	LOC, PTA 8 hours	113	38	14	22
Pt11	39		2	“Brief” LOC	72	28	42	8
Pt12	19		10	LOC unknown length	104	31	63	20
Pt13	30		11	Coma 2 weeks	91	40	49	3
Pt14	49		2	Collapsed with seizure	111	49	61	5

Note: M = mean; SD = standard deviation; GCS = Glasgow Coma Scale; LOC = loss of consciousness; PTA = post traumatic amnesia; ACRM = American Congress of Rehabilitation Medicine; TBI, traumatic brain injury.

## 3. Results

### 3.1. Boredom Proneness

Analyses of BPS scores across the three groups were conducted using a one-way analysis of covariance (ANCOVA), with participant’s age as a covariate, given that the moderate-to-severe TBI group was significantly older than the other two groups (which did not differ from one another). Results indicated that the relationship between age and BPS scores did not differ significantly across each group. That is, there was no significant interaction between age and group on the total BPS score [*F*(2, 134) = 1.52, *p* = 0.222], the internal stimulation subscale score [*F*(2, 132) = 2.26, *p* = 0.109], or the external stimulation subscale score [*F*(2, 132) = 2.47, *p* = 0.089]. Thus, the interaction term was removed from subsequent models analyzing BPS scores.

Numerically, the BPS scores were highest for the moderate-to-severe TBI group, next highest for the mild TBI group and lowest for controls (Table 1). Independent samples’ *t*-tests contrasting BPS scores for each group showed that the mild and moderate-to-severe TBI patients did not differ from one another, and controls did not differ from the mild TBI group. The moderate-to-severe TBI patients did have somewhat higher BPS scores when compared with controls (*t*(20.63) = 1.94, *p* = 0.03; one tailed, unequal variance assumed). Scores on the individual subscales of the BPS did not differ significantly across the three groups. 

### 3.2. Depression

ANCOVA was performed on the BDI-II scores across the three participant groups, controlling for age. Results yielded a significant interaction between participant group and age [*F*(2, 134) = 4.05, *p* < 0.05]. Linear regression lines fit to the data yielded significant *positive* slopes relating age and BDI-II scores in both the control and mild TBI groups and a non-significant *negative* slope in the moderate-to-severe TBI group. Multiple regression analysis indicated that for the control and mild TBI groups, increased age was associated with higher levels of depressive symptoms. In contrast, for the moderate-to-severe TBI group, there was no such association. 

### 3.3. Relationship between Boredom and Depression

Bi-variate correlations were performed between measures of boredom (BPS) and depression (BDI-II) to explore the relationship between the two constructs across the participant groups. Results revealed significant correlations between BPS and BDI-II scores in all three groups (controls: *r* = 0.409, *p* < 0.01; mild TBI: *r* = 0.616, *p* < 0.01; moderate-to-severe TBI: *r*= 0.764, *p* < 0.01). The correlations were then contrasted across the groups using z-scores [19]. This analysis revealed a trend towards a stronger relationship between boredom and depression in the mild TBI (*z* = 1.41, *p* = 0.15) and moderate-to-severe TBI groups (*z* = 1.78, *p* = 0.07), when each was directly contrasted with controls. 

**Figure 1:**
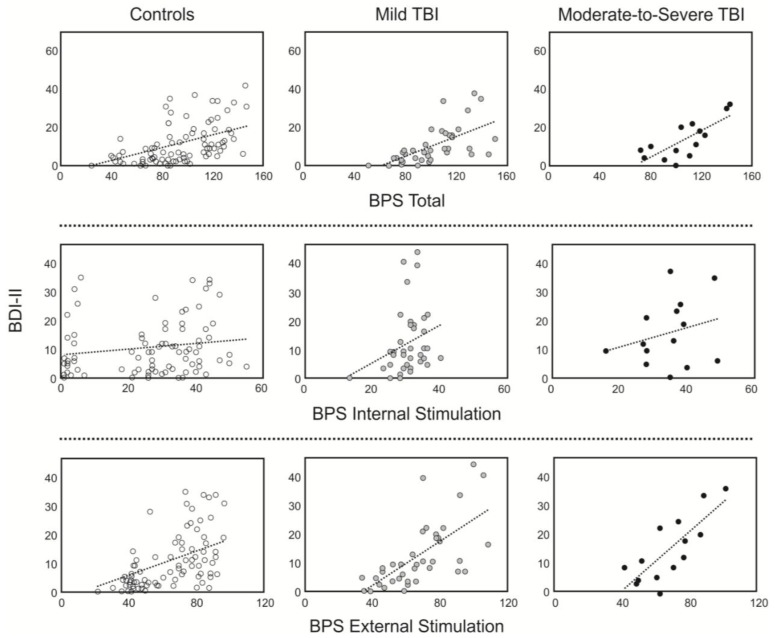
Scatterplots showing the relationship between the boredom proneness (BPS; x-axis) and depressive symptoms (BDI-II; y-axis). BDI-II = Beck Depression Inventory; BPS = Boredom Proneness Scale.

When the BDI-II was correlated with the individual subscales of the BPS, no significant relationships were found between the internal stimulation subscale items and the BDI-II scores in any of the groups (controls: *r* = 0.134, *p* = 0.218; mild TBI: *r* = 0.268, *p* = 0.104; moderate-to-severe TBI: *r* = 0.260, *p* = 0.369). In contrast, the external subscale was significantly correlated in all three groups (controls: *r* = 0.393, *p* < 0.001; mild TBI: *r* = 0.662, *p* < 0.001; moderate-to-severe TBI: *r* = 0.786, *p* < 0.001). This relationship was not significantly different across the mild and moderate-to-severe TBI groups. However, there was a significant difference between the moderate-to-severe TBI group and controls (*z* = −2.01, *p* < 0.05), and the difference between the mild TBI and control groups approached significance (*z* = 1.89, *p* = 0.057). Thus, the relationship between depressive symptoms and the external stimulation subscale of the BPS was greater in the mild and moderate-to-severe TBI groups than controls (Figure 1). 

Given these results, we reanalyzed our larger sample of healthy individuals from our previous work (*n* = 823; males = 243, M age = 20 years, SD = 3.7) [6] to determine the extent to which the need for external stimulation accounted for the relationship between boredom and depression in a larger sample. Correlations indicated that the external stimulation subscale was more highly correlated with the BDI-II (*r* = 0.527, *p* < 0.001) than was the internal stimulation subscale (*r* = 0.321, *p* < 0.001; *z* = −6.50, *p* < 0.001) [6]. Moreover, the squared correlation coefficients (*r*^2^) revealed that 28% (*p* < 0.001) of the variance in the BDI-II was attributable to the external stimulation subscale, while only 10% (*p* < 0.001) was attributable to the internal stimulation subscale. This supports the notion that the need for external stimulation is the driving force behind the relationship between boredom proneness and depression in our larger sample—a relationship that is magnified following TBI.

## 4. Discussion

We investigated the relationship between depression and boredom in patients with varying degrees of TBI, where boredom has been reported to be pervasive [1,2,4]. All three groups evidenced a strong correlation between boredom and depression, as shown in previous work (Figure 1) [6,7]. This relationship warrants further investigation. Our earlier work showed that the two constructs do indeed represent distinct affective states and that any commonalities in the items on each scale cannot account for the strong relationship [6]. In addition, at least one study looking at the effects of pharmacological treatment (citalopram) for depression in cancer patients [20] found that depressive and boredom symptoms ameliorate along different time scales (depressive symptoms are alleviated more rapidly than self-reported boredom). This provides further support to the notion that the two constructs tap into different subjective experiences and that boredom does not simply reflect a symptom or epiphenomenon of depression. Regardless, it is not clear at this stage what underlying mechanisms may be common to the two experiences and what, if any, the causal relationship is between the two. It is also worth pointing out that depression and boredom may manifest in distinct ways in different patient populations. 

It was perhaps surprising that there was not a more stark difference in boredom proneness in the TBI groups given the documented subjective reports of elevated boredom in TBI [1,4]. It may be the case that the BPS lacks sensitivity with this population. In addition, TBI patients may, in fact, show elevated levels of boredom proneness *relative* to premorbid levels, which we were not able to measure here. In addition, many of our moderate-to-severe TBI patients had suffered their injury many years prior to testing (Table 1). Although this represents a highly speculative conjecture, it may well be the case that boredom levels subside with age, in general, and with time post injury, in particular, both possibilities that require further research. 

The control and mild-TBI groups showed a relationship between depression and age, such that depressive symptoms *increased* with age. One would have expected to see a similar pattern in the moderate-to-severe TBI group, especially if one considers that in this population, depressive symptoms will be likely to increase with increasing insight into the patient’s condition. Therefore, while initially lower levels of insight may prove to be prophylactic for depression in TBI [4], as insight is gained, depressive symptoms should be more likely to appear. Further research is needed to determine the course of depressive symptoms in TBI. The small sample size here prevents us from making any reasonable speculation about the lack of an association between depression and age in the moderate-to-severe TBI group.

When full BPS scores were considered, results showed a trend towards a stronger relationship between boredom and depression in the mild and moderate-to-severe TBI groups than in controls. This is not surprising, given the high incidence of post-injury depression in patient groups [4,21]. In all three groups, the relationship between boredom and depression was largely driven by the perceived need for external stimulation from the environment. This relationship was stronger in both TBI groups when contrasted against controls, although the difference only approached significance for the mild TBI group. This failure to satisfy a need for external stimulation could plausibly lead to both boredom and depression simultaneously. Given our prior work showing that boredom and depression are indeed two distinct affective states [6], along with other work showing that they respond to pharmacological treatment along different time scales [20], it is nevertheless plausible that boredom and depression can be experienced independently and that the failure to satisfy a need for external stimulation could lead to one or either affective state in isolation. As such, it remains an open question as to why a perceived need for external stimulation—and presumably the failure to satisfy that need—could lead to boredom in one instance and depression in another? This is perhaps particularly pertinent when taking into consideration one of the cardinal difficulties of patients with depression, who commonly suffer from recursive negative self-evaluations that, on face value, are *internal* evaluations [18]. Although completely speculative at this stage, two potential explanations can be considered; first, the failure to garner satisfactory stimulation from the environment may be *interpreted* in distinct ways by bored *vs.* depressed individuals. The bored individual, particularly those who see the experience as aggressively dissatisfying [8,10], may attribute the failure to be stimulated as external to themselves—that is, there is something wrong with the world. In contrast, the depressed individual may interpret the same experience as an internal failure—there is something wrong with me. This, of course, requires further research to examine the cognitive appraisal and attribution styles of both depressed and bored individuals (see [22,23,24] for a discussion of coping styles and depression following brain injury). The second possibility is that depression and boredom follow one from one another in a temporal manner, with depression arising only after prolonged boredom or disengagement from the environment [12], both of which are known to cause feelings of hopelessness and helplessness—hallmark features of depression [25,26]. Of course, the opposite temporal sequence could also occur, with boredom following on as a consequence of depressive symptoms or episodes. Clearly, further research is required to longitudinally investigate the time course of boredom and depression, especially in TBI patients. 

The findings presented here may have implications for the treatment of depression, in general, and in patients who have suffered brain injury, in particular. One popular therapeutic approach to the treatment of depression is behavioural activation therapy [27], which attempts to engage the patient in the pursuit of a hierarchy of self-generated goals with rewards given for success. Boredom, as a stand-alone affective experience [6], may represent a serious impediment to this approach. That is, the agitated boredom prone individual [8,10] is, by definition, *already* motivated to engage in activities, but fails to see or experience the pleasure in doing so. In the context of behavioural activation therapy, asking such a patient to engage *more* may be futile. Instead, it may be more important to address the disconnection between the individual’s high level of motivation to engage with the environment and the perceived failure to satisfy those desires. As such, a therapeutic approach that focuses more on cognitive appraisal to realistically experience the environment may be more successful in the treatment of depression in patients who are prone to agitated boredom. 

Why boredom and depression are more strongly correlated in patients with TBI should be the focus of further research. It may simply be that reduced cognitive resources in TBI mean that these patients are more vulnerable to both affective states. Alternatively, one brain region commonly affected in TBI, and known to be dysfunctional in patients with depression, is the orbitofrontal cortex (e.g., [28]). This brain region is known to be important for representing the reward value of stimuli [29]. A failure to accurately register or respond to external reinforcers may play a significant role in both boredom and depression. With the relatively small samples sizes tested here, coupled with the fact that our groups were not matched on key demographics (*i.e.*, age and education) and there was, at least in the moderate-to-severe TBI group, a long time post injury, it is difficult to address these specific hypotheses. 

## 5. Conclusions

What the current data show is that the relationship between boredom and depression is strongest in patients suffering moderate-to-severe TBI and is driven by a failure to feel stimulated by the external environment. Important future steps in this research include following the evolution of each affective state over time in TBI, as well as employing both structural (e.g., Diffusion Tensor Imaging) and functional neuroimaging techniques to determine the underlying neural bases of boredom and depression in this population.
